# Inflammatory Mediators in Vascular Disease: Identifying Promising Targets for Intracranial Aneurysm Research

**DOI:** 10.1155/2015/896283

**Published:** 2015-04-01

**Authors:** David M. Sawyer, Peter S. Amenta, Ricky Medel, Aaron S. Dumont

**Affiliations:** ^1^Tulane University School of Medicine, 1430 Tulane Avenue, New Orleans, LA 70112, USA; ^2^Department of Neurosurgery, Tulane University School of Medicine, 131 S. Robertson Street, Suite 1300, No. 8047, New Orleans, LA 70112, USA

## Abstract

Inflammatory processes are implicated in many diseases of the vasculature and have been shown to play a key role in the formation of intracranial aneurysms (IAs). Although the specific mechanisms underlying these processes have been thoroughly investigated in related pathologies, such as atherosclerosis, there remains a paucity of information regarding the immunopathology of IA. Cells such as macrophages and lymphocytes and their effector molecules have been suggested to be players in IA, but their specific interactions and the role of other components of the inflammatory response have yet to be determined. Drawing parallels between the pathogenesis of IA and other vascular disorders could provide a roadmap for developing a mechanistic understanding of the immunopathology of IA and uncovering useful targets for therapeutic intervention. Future research should address the presence and function of leukocyte subsets, mechanisms of leukocyte recruitment and activation, and the role of damage-associated molecular patterns in IA.

## 1. Introduction

Despite advances in surgical and medical management, aneurysmal subarachnoid hemorrhage (aSAH) continues to result in significant morbidity and mortality [1]. Although aSAH is devastating, it is rare, occurring in approximately 1% of patients harboring an aneurysm per year [2]. The discovery of an unruptured IA can therefore present a clinical dilemma, with patient and physician weighing the risk of microsurgical or endovascular intervention against the natural history of the disease [3]. As a result, there is a strong need for the development of noninvasive therapies for use in the treatment of patients found to have unruptured IA, or those who are at high risk for developing the disease.

A concerted research effort has been mounted with the goal of identifying the mechanisms that underlie IA formation and rupture. Aneurysm samples collected from human patients during treatment have been examined, providing many clues to the pathology of the disease. However, the majority of experimental studies have been performed in animal models, allowing for the manipulation of specific variables and the observation of the progression of IA. For clarity, it is clearly stated in this paper whether findings regarding IA are the result of human or animal studies.

IA formation is characterized by a distinct and consistent set of changes in the architecture of the arterial wall. Animal models have suggested that loss of the internal elastic lamina (IEL) occurs early in the course of the disease, followed by hyperplasia of the tunica intima and disarray of vascular smooth muscle cells (VSMCs) in the tunica media [4]. Additionally, there is a general decrease in cellularity of the tissue, along with changes in the extracellular matrix composition. Collagen I and fibronectin are distributed ectopically, and the normal predominance of collagen III/IV and laminin is decreased [5, 6]. Functionally, key features of IA demonstrated in animal models include endothelial dysfunction with activation of the vascular endothelium [7], as well as VSMC apoptosis and modulation of VSMCs to a phenotype polarized toward extracelluar matrix (ECM) remodeling and inflammatory signaling [4]. The natural history of IA does not appear to be homogenous; the processes of aneurysm formation, progression, and rupture are likely driven by different mechanisms [8]. Recent findings support the notion that rupture in particular is a distinct event, which has important implications for the development of therapeutic strategies [9, 10]. Currently, many of the pathologic changes underlying IA are thought to be the result of chronic inflammation in the vessel wall, but our knowledge of the mediators and processes involved remains fragmented.

The pathobiological characteristics of IA share many similarities with atherosclerosis; indeed, the two entities appear to overlap to a significant extent. One of the key features of atherosclerotic lesions is the accumulation and oxidation of lipids in the vascular wall, and this process has been well demonstrated in IA. Tulamo et al. first reported the presence of oxidized low-density lipoproteins (OxLDLs) in the walls of aneurysms from human patients [11]. Later work by Frösen et al. further characterized this process, showing that accumulated neutral lipids are taken up by cells such as VSMCs, are oxidized, and produce inflammation and a humoral immune response in human IA walls [12]. Importantly, this study associated these features with degeneration of the vessel wall and aneurysm rupture. A recent review by the same group discusses the presence of atherosclerotic lesions in IAs and the importance of lipid deposition in the progression of the disease [13]. Other studies of human IA tissue have noted the presence of additional atherosclerotic changes such as smooth muscle cell proliferation, macrophage and lymphocyte infiltration, and neointima formation [14, 15]. The similarities in histopathologic appearance, along with shared risk factors, suggest that the mechanisms underlying the pathogenesis of IA and atherosclerosis are likely fundamentally similar [4]. Working from this hypothesis, this paper will present a focused review of the literature related to the role of inflammatory mediators in vascular disease and intracranial aneurysms, with the goal of highlighting promising avenues of investigation in IA. Special attention will be paid to pro- and anti-inflammatory subsets of leukocytes, which must be delineated in order to allow for their therapeutic modulation.

## 2. Macrophages

The presence of macrophages in diseased vasculature has been shown to be a central characteristic of both IA and atherosclerosis. Macrophages have been repeatedly identified in tissue samples taken from patients with IA, represent the majority of the leukocytic infiltrates, and are associated with the risk of rupture [16–18]. Macrophages have been extensively characterized in the setting of atherosclerosis, and a number of well-designed studies have addressed their role in the formation of aneurysms.

Kanematsu et al. replicated the histologic characteristics of human aneurysms in a mouse model of IA, showing robust leukocyte infiltration into aneurysm walls composed primarily of macrophages [19]. Furthermore, they demonstrated that macrophage depletion by clodronate liposome treatment greatly reduced the incidence of aneurysms. The mechanism by which macrophages participate in IA pathogenesis can be broadly divided into two categories: remodeling of the ECM and elaboration of inflammatory cytokines.

### 2.1. Macrophage Contributions to Extracellular Matrix Remodeling

Early evidence that ECM remodeling was important in the pathogenesis of IAs was provided by the examination of proteinases in human aneurysm tissue specimens retrieved by surgical resection [20, 21]. Using* in situ* zymography, it was found that the activity of serine proteases and matrix metalloproteinases (MMPs) was elevated in aneurysms, compared to control vessels. Immunohistochemical localization showed that the particular enzymes associated with aneurysms were plasmin, MMP-2, MMP-9, and MT1-MMP (an activator of MMP2) [22]. Later work in rat and mouse models of IA indicated that macrophage infiltration and MMP expression were associated with aneurysm formation, with specific demonstration of MMP-2 and MMP-9 activity [23, 24]. Furthermore, tolylsam, a selective inhibitor of MMP-2, -9, and -12, reduced the progression of aneurysms to an advanced stage in the model [23]. Other reports utilizing mouse models have confirmed the presence of high MMP activity in IA, as well as attenuation of aneurysm formation with MMP blockade or knockout [19, 25]. IEL loss and ECM remodeling are features of IA that are likely to be mediated by MMPs derived from macrophages and VSMCs, leading to the loss of vessel wall integrity that is characteristic of the disease.

Similar roles for proteolytic enzyme activity have been established in atherosclerosis. In this setting, two distinct pathologic processes have been linked to MMP expression. First, outward remodeling of the vessel wall is observed in the presence of atherosclerotic plaques, which is driven by the degradation of elastin and collagen [26]. This increase in vessel diameter is mediated by proteolysis [27] and could be considered analogous to the early stages of aneurysm formation. Later, in more advanced atherosclerotic lesions, collagenase activity promotes thinning of the plaque's fibrous cap, an event that precedes plaque rupture and is the cause of most thrombotic phenomena in myocardial infarction and ischemic stroke [28]. Some evidence exists to suggest that macrophages play a similar role in the rupture of IA; one study found that inhibition of MMPs with tetracycline stabilized IAs in mice after they had formed, preventing or delaying rupture [29].

### 2.2. Macrophage Promotion of Inflammation

In addition to their direct effects on ECM remodeling, macrophages are key participants in the elaboration of proinflammatory signaling. Macrophages secrete a number of factors that have been implicated in IA genesis, including tumor necrosis factor alpha (TNF-*α*), interleukin-1 beta (IL-1*β*), and IL-6 [4].

TNF-*α* in particular has been well characterized as a mediator of IA pathogenesis. A number of single-nucleotide polymorphisms (SNPs) in the gene for TNF-*α* have been identified as risk factors for IA or SAH in patients [30], and human aneurysm specimens display increased expression of the molecule [31]. Increased serum levels of TNF-*α* are associated with aSAH in patients [32], and TNF-*α* signaling has been implicated in IA formation and rupture by a large genome-wide association study [33]. TNF-*α* is known to act on two distinct receptors: TNFR1, which is expressed constitutively and binds soluble TNF-*α*, and TNFR2, which is expressed on endothelial and immune cells and is acted on by membrane-bound TNF-*α* [30]. A recent study by Aoki et al. demonstrated that TNFR1 is expressed in rodent intracranial arteries and that deficiency of this receptor markedly diminished IA formation in a mouse model [34]. Any potential contribution by signaling through TNFR2 remains to be determined.

Experimental work in animals has shown that TNF-*α* is released in aneurysm walls, induces inflammatory cytokine expression and macrophage infiltration [34], and drives both the formation and the rupture of IA [35]. Additionally, TNF-*α* causes changes in the phenotype of VSMCs. Signaling through Kruppel-like transcription factor 4 (KLF-4), TNF-*α* reduces production of contractile proteins and induces expression of proinflammatory molecules (including MMPs, MCP-1, and IL-1*β*)* in vitro* [36, 37]. However, these phenotypically modified VSMCs may also play a protective role against aneurysm rupture, as they are responsible for the intimal hyperplasia that likely serves to occlude early aneurysm lumens [38].

The loss of functional VSMCs is a characteristic feature of IA that results in the inability to replenish the components of the ECM and is thus strongly associated with disease progression and rupture [38]. A recent study by Marbacher et al. demonstrated that depletion of mural cells from abdominal aortic aneurysms in rats leads to aneurysm growth and rupture, likely due to the lost ability to form an occluding thrombus [39]. The same group showed that transplantation of VSMCs into decellularized, embolized rat aneurysms restored the function of the intraluminal thrombus and protected against wall degradation and rupture [40]. These results highlight the important role that VSMCs play in preventing the progression of aneurysmal changes. Although necrosis and migration/phenotypic modulation may contribute to the loss of VSMC cellularity in IA, a major contributor to this process appears to be apoptosis [38]. In the early stages of IA formation in mice, VSMC apoptosis seems to be driven by inflammatory processes, including the release of macrophage cytokines such as IL-1*β* [41]. However, further apoptosis leading to wall degeneration and rupture is not associated with cytokine signaling in human tissue and may rather be the result of oxidative stress or lipid accumulation [38, 42]. TNF-*α* causes diminished VSMC cellularity via apoptosis in atherosclerosis [43], but this effect has not been demonstrated in IA. TNF-*α* induces apoptosis via signaling through Fas-associated death domain (FADD) and caspase-8 [44]. Although both TNF-*α* and FADD are increased in human aneurysms [31], a more robust study of human tissue by Laaksamo et al. found no evidence of activation of caspase-8, indicating that apoptosis likely does not occur by extrinsic TNF-*α* signaling [42].

Taken as a whole, these findings call for a more thorough investigation of the mechanisms by which macrophage-associated inflammatory signaling contributes to IA and whether interruption of this signaling could prove to be a viable therapeutic approach.

Subpopulations of macrophages do exist, although their role in inflammatory vascular disease is only now coming to light. Recent research has identified two groups of activated macrophages with distinct functions that are polarized by the inflammatory signaling milieu. M1 macrophages are proinflammatory cells that are activated by interferon gamma (IFN-*γ*) and which produce reactive oxygen/nitrogen species and elaborate IL-1*β*, TNF-*α*, and IL-6 [45]. In contrast, M2 macrophages are involved in the resolution of inflammation and repair of tissue. They are activated by IL-4 and IL-13 likely produced by T helper-2 cells via the nuclear receptor PPAR*γ*, which also suppresses NF-*κ*B activity in these cells [46]. Products of M2 macrophages are anti-inflammatory and include IL-10, TGF-*β*, and IL-1 receptor antagonist (IL-1Ra) [45, 46].

Both M1 and M2 macrophages have been identified in IAs from human patients, and a predominance of M1 polarization is associated with rupture [47]. This is consistent with the previously demonstrated importance of IL-1*β*, TNF-*α*, and IL-6 in aneurysm pathogenesis. Similarly, advanced atherosclerotic lesions exhibit a high relative proportion of M1 cells, with models of plaque healing characterized by elevated numbers of M2 cells [26]. However, these findings do not fully describe the range of functions that activated macrophages can exert* in vivo*, and further research is needed to determine the potentially contradictory roles of these cells in both diseases [48].

### 2.3. Macrophage Recruitment and Activation

As macrophages are likely to be early mediators of the inflammatory cascade in IA, it is important to understand the mechanisms by which they are recruited into the vascular tissue and activated. In both aneurysm and atherosclerosis, the key initiating factor appears to be activation of the endothelium and subsequent endothelial dysfunction. Patterns of hemodynamic shear stress play a role in activating the endothelium in both diseases [7, 49–52], accounting for the preferential formation of aneurysms and plaques at areas of turbulent blood flow, such as arterial bifurcations. Lipid metabolism and accumulation are additional inducers of endothelial activation that have been identified in atherosclerosis [53–55].

Some of the mechanisms by which endothelial cells participate in the recruitment of macrophages to the site of an IA have been uncovered, and related research in the realm of atherosclerosis may provide clues for further investigation. The chemokine monocyte chemoattractant protein 1 (MCP-1) has been shown to be expressed in human aneurysm specimens [19]. Its causal role has been investigated in mouse models utilizing MCP-1 blockade and knockout, where disruption of MCP-1 signaling is associated with decreased macrophage accumulation, MMP expression, and aneurysm formation [56]. Expression of MCP-1 as a feature of endothelial activation appears to be induced by signaling through the transcription factor NF-*κ*B, which is induced by hemodynamic shear stress [57]. Aoki et al. reported that NF-*κ*B is highly upregulated in the walls of human IAs and that knockout or blockade of the transcription factor in animal models resulted in decreased aneurysm formation [58]. Furthermore, NF-*κ*B colocalized with endothelial cells expressing MCP-1 in this study.

NF-*κ*B and its induction of MCP-1 have likewise been implicated in the recruitment of macrophages to atherosclerotic plaques [59, 60], with additional research demonstrating the importance of upregulation of cellular adhesion molecules. Classical extravasation of macrophages has been demonstrated in atherosclerosis, mediated by selectins/selectin-ligands and integrins/cellular adhesion molecules [48]. Aoki et al. tied the expression of the adhesion molecule VCAM-1 to NF-*κ*B signaling in rat aneurysms [58], but no study has comprehensively examined the behavior of endothelial cellular adhesion molecules and the process of leukocyte extravasation in IA.

## 3. Antigen-Presenting Cells

An additional aspect of macrophage function that has received little attention in the IA literature is the detection of molecular signals of cellular injury and death, known as damage-associated molecular patterns (DAMPs). Classically, these signals are represented by the subclass pathogen-associated molecular patterns (PAMPs), a set of conserved biochemical features of microbes that are detected primarily via toll-like receptors (TLRs) and which induce an immune response through NF-*κ*B signaling [61]. However, further research has revealed that the DAMP pathway can be activated by endogenous molecules whose release or modification signals trauma to cells [61].

In the setting of atherosclerosis, DAMPs have been readily identified and shown to play a key role in recruiting inflammation. Specifically, a class of molecules known as oxidation-specific epitopes (OSEs) has been implicated. These are host-derived molecules that have been altered by reactive oxygen species, and they are thought to be one of the early initiators of inflammation in atherosclerosis [62]. A common manifestation of the OSE is in the form of OxLDLs, which have been clearly demonstrated to accumulate in human IA [11, 12]. Studies of atherosclerosis have shown that OxLDLs are recognized by macrophage pattern-recognition receptors (PRRs) such as CD36 and TLR-4 [62] and promote proinflammatory signaling, such as TNF-*α* [26, 30] and leukocyte recruitment [62]. Dendritic cells, which are antigen-presenting cells (APCs), also play an important role in response to the presence of OSEs. Dendritic cells are important drivers of the maturation and polarization of T cells and thus represent an important link between the innate and adaptive immune systems in atherosclerotic disease [63].

More evidence for the importance of DAMP recognition in IA has been provided by genome-wide association studies conducted using tissue from human patients. Kurki et al. found that TLR signaling is one of the processes strongly associated with ruptured aneurysms [33]. Additionally, a similar but smaller study by Krischek et al. found that the major ontological group of differentially expressed genes corresponded to the function of “antigen processing” [64]. Follow-up using real-time polymerase chain reaction and immunohistochemical localization led the authors to conclude that APCs, specifically monocytes and macrophages expressing major histocompatibility complex II (MHCII), perform an important function in the pathogenesis of aneurysms.

**Figure 1:**
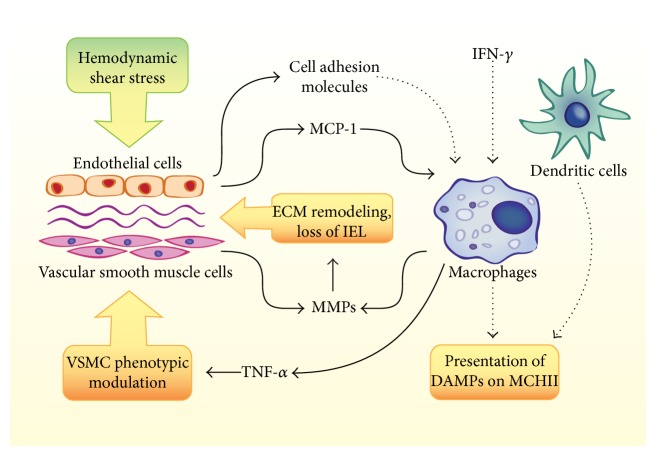
Summary of known and hypothesized contributions of macrophages and dendritic cells to intracranial aneurysm (IA). Interactions that have been demonstrated in IA models are depicted with solid arrows. Additional mechanisms that may be hypothesized based on other vascular inflammatory diseases are depicted with dotted arrows. Hemodynamic shear stress contributes to the recruitment of macrophages to the aneurysm wall via expression of MCP-1, a process that may also be aided by cell adhesion molecules. Macrophages are likely activated by IFN-*γ* and have been shown to contribute to IA by the secretion of TNF-*α* and MMPs. Additionally, macrophages and dendritic cells could contribute to inflammation via the presentation of DAMPs (such as oxidized lipids) on MHCII.

Macrophages and dendritic cells clearly exhibit a strong influence on the pathogenesis of IA, and their specific contributions should be more fully explored.  Figure 1 highlights the roles that have been demonstrated and hypothesized for macrophages and dendritic cells in IA. Potential lines of future research include strategies for reducing ECM remodeling, blockade of proinflammatory signaling, and prevention of macrophage recruitment and activation.

## 4. Lymphocytes

Studies of human aneurysm tissue collected during surgical resection have provided evidence for the involvement of lymphocytes in the generation of IA. Infiltrates of T cells and more rarely B cells have been described in aneurysm walls [16]. Greater numbers of lymphocytes are associated with aneurysms that have ruptured [17] and these cells are preferentially located in tissue around the area of rupture [14]. Despite these observations, it remains undetermined whether lymphocytes play a causal role in the formation of aneurysms or whether they merely represent a response to tissue injury.

**Figure 2:**
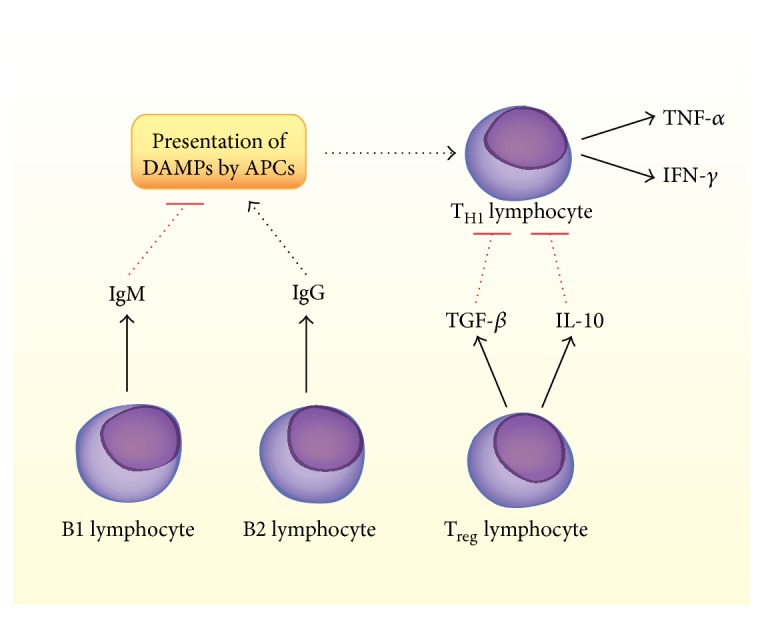
Summary of potential contributions by lymphocytes to IA. T- and B-lymphocytes have been identified in human IA tissue specimens, but their functions and the presence of subsets of these cells are not well characterized. IgM and IgG are present in IA, though specific populations of B1 and B2 lymphocytes have not been confirmed as the source of these immunoglobulins. Additionally, although IgG plays a proinflammatory role and IgM plays an anti-inflammatory role in atherosclerosis, these strict characterizations have not been borne out in models of IA. T_H1_ lymphocytes, which express TNF-*α* and IFN-*γ*, may be present in IA and could be activated by DAMP presentation by macrophages and dendritic cells. TNF-*α* plays an important role in IA pathogenesis. T_reg_ lymphocytes may be present and could antagonize T_H1_ cells through the actions of TGF-*β* and IL-10.

Furthermore, little is known about the presence or role of the many subsets of lymphocytes in aneurysms. Important and widely differentiated effects have been attributed to CD4+ T helper (T_H_) cells, T regulatory (T_reg_) cells, and subpopulations of B-cells (B_1_ and B_2_) in atherosclerotic disease, and their potential contributions to IA should be investigated.  Figure 2 outlines the roles that some of the subclasses of lymphocytes could play in the progression of IA.

## 5. T Cells

Helper T cells can be further subdivided into T_H1_ and T_H2_ cells, characterized by their differing cell-surface markers and secreted cytokines. Classically, these subgroups are associated with cellular and humoral immunity, respectively [65]. T_H1_ cells are disproportionately represented in atherosclerotic plaques and elaborate the soluble factors IFN-*γ* and TNF-*α*, resulting in proinflammatory and proatherogenic effects [66]. As previously described, TNF-*α* plays a vital and well-characterized role in the pathogenesis of IA, suggesting that T_H1_ cells may be involved in mediating the disease. IFN-gamma has not been studied in the setting of IA but is a key effector of macrophage and plasma B-cell activation and a promoter of endothelial adhesion molecules [67, 68]. Conversely, T_H2_ cells are found less commonly in plaques, and their function in atherosclerosis is ill-defined and controversial [67].

Regulatory T cells are important modulators of the immune system, responsible for maintaining tolerance to self-antigen and abrogating autoimmune disease. In general, T_reg_ cells antagonize the effects of T_H_ cells through the secretion of IL-10 and transforming growth factor beta (TGF-*β*) [66]. In the setting of atherosclerosis, these cells predictably confer a protective benefit against plaque formation. Experiments have shown increased atherogenesis in mouse models characterized by depletion of T_reg_ cells or blockade of IL-10 and TGF-*β* [69, 70]. No studies have assessed the function of T_reg_ cells or IL-10 in IA, though TGF-*β* signaling has interestingly been associated with both protective and pathologic effects in aneurysms from human patients [71–73].

## 6. B Cells

B-lymphocytes are likewise implicated in atherosclerosis and consist of subgroups with differential effects on disease progression. A basic distinction can be made between B1 cells, which are primarily found in the lungs and peritoneum, and B2 cells, which are prominent in lymphatic tissue and the spleen [74].

B2 cells may have a proinflammatory effect. Experiments involving selective depletion of B2 populations via anti-CD20 antibodies and BAFFR knockouts have shown decreased plaque burden in mice [75–77]. Additionally, transfer of B2 cells into lymphocyte-deficient mice induces atherosclerosis, further suggesting that these cells contribute to atherogenic inflammation [78]. Multiple hypotheses exist to explain this effect, but of particular interest is the involvement of IgG and IgE antibodies, which have been associated with cardiovascular disease [79, 80]. IgG may facilitate macrophage-mediated uptake of OSEs and damage to endothelial cells, and IgE could play a role in destabilizing plaques [81]. Production of IgG and IgE is selectively accomplished by the B2 subpopulation and requires antibody class switching to occur under the influence of T-cell cytokines such as IL-4 and IFN-*γ* [74].

Conversely, the B1 subpopulation has been shown to exhibit a clear atheroprotective effect, through well-characterized mechanisms. In contrast to the dependence of IgG and IgE production on inflammatory cytokine signaling, B1 cells produce predominantly IgM via a T-cell-independent process [81]. A dominant portion of the IgM secreted by these cells is natural antibody (NAb) that possesses specificity to OSEs and mediates the removal of OxLDL and apoptotic cells from tissue [82–84]. This clearance of debris blocks the proinflammatory effects of the OSEs and prevents the formation of foam cells, abrogating the process of atherogenesis in animal models [81].

Although the importance of B cells in IA is not well characterized, multiple studies have implicated humoral immunity in the pathology of aneurysms. Deposition of IgG and IgM in the walls of human IA specimens has been identified [11], and the presence of antibodies specific for oxidized lipids has likewise been shown [12]. Interestingly, these studies question the characterization of IgG as pathologic and IgM as protective. Frösen et al. showed that serum levels of anti-lipid IgG were in fact higher in patients who had unruptured IAs, compared to those with rupture and SAH [12]. The plasma levels of IgM showed a similar but nonsignificant trend. Furthermore, there is lack of evidence showing that deposition of immunoglobulin in IA walls is correlated with the infiltration of leukocytes [13], contrary to what may be hypothesized from the atherosclerosis literature. Further research is needed to determine the influence of B cells and humoral immunity on the processes underlying IA formation and rupture.

## 7. Discussion

A wealth of recent research has characterized IA as a disease driven primarily by inflammatory processes occurring in the walls of cerebral vessels. Although many well-designed studies have addressed the roles of individual inflammatory mediators in IA, the field lacks a comprehensive understanding of its immunopathology. Many parallels have been drawn between IA and atherosclerotic disease, and the large volume of literature produced in the context of the latter could provide a roadmap for future investigation.

Table 1 presents a simplified view of some of the known and potential contributors to inflammation in the context of IA. It is evident that there are some key recurring factors that are likely to play important roles in the immunopathology of the disease. Interferon gamma produced by T_H1_ cells is an activator of both M1 macrophages and B2 lymphocytes and the proinflammatory subsets of those respective cell types. Thus, interferon gamma represents a potential therapeutic target for the suppression of inflammation [67]. Similarly, IL-4 and IL-13 elaborated by T_H2_ cells are capable of activating M2 macrophages involved in tissue repair and the resolution of inflammation [46]. Although T_H2_ cytokines are not commonly present in vascular inflammatory lesions [67], these factors could represent a strategy for polarizing the lymphocyte and macrophage populations toward anti-inflammatory subsets. There is evidence from models of atherosclerosis that identifying and targeting these reparative cells could even allow healing of the pathologic features of vascular inflammatory disease [26].

In addition to the contributions of these cell types to the formation and rupture of aneurysms, functional questions remain to be addressed. The process of leukocyte recruitment and extravasation should be thoroughly characterized in IA, as it represents a link between the known features of endothelial activation and inflammatory infiltration. Therapeutic intervention could potentially block this key stage in the disease, as demonstrated in previous animal studies [85, 86]. Additionally, the role of damage-associated molecular patterns in the pathogenesis of IA should be investigated. Due to their importance in the activation of macrophages and T-lymphocytes (via dendritic cells), DAMPs are likely to be involved in aneurysm formation. However, the identity of these molecules and the specific actions they exert are unknown. Furthermore, therapeutic manipulation of B-lymphocyte subsets may allow regulation of the macrophage reaction to DAMPs [74]. While B2 cells promote proinflammatory macrophage uptake of DAMPs, natural antibodies produced by B1 cells neutralize these molecules and allow them to be cleared without an inflammatory response [84].

Exploring these possibilities will require a concerted multidisciplinary research effort. Animal models will remain important, allowing investigators to block or deplete specific cell populations or signaling factors. Actions of inflammatory mediators could also be studied via selective reconstitution of cells and/or molecules into immune-deficient animals [87].* In vitro* work could also provide important insight. In particular, coculture techniques hold promise in the demonstration of interactions between endothelial cells, VSMCs, and leukocytes in a controlled environment [88]. Finally, the prevalent use of anti-inflammatory medications by the general population provides an opportunity for the investigation of their effects on aneurysm formation and rupture in large human cohorts [89].

## 8. Conclusions

IA is an important disease that causes significant mortality and morbidity, and for which a noninvasive therapeutic option is currently unavailable. It is clear that inflammation is a key factor underlying the pathogenesis of aneurysms, and elucidating the mechanisms involved could uncover targets for the development of better treatments. A focused and sustained research effort will be necessary to fully characterize the immunopathology of IA. Intracranial aneurysm seems to share features with other inflammatory diseases of the vasculature, and insight may be gained from comparison and contrast of these disorders.

## Figures and Tables

**Table 1:** A summary of some known and hypothesized inflammatory mediators with roles in intracranial aneurysm. This table provides an outline of possible avenues of investigation for further research into the immunopathology of IA.

Inflammatory targets in intracranial aneurysm				
Cell type	Activators	Effectors	Function	Targets for intracranial aneurysm

M1 macrophages	IFN-*γ*, NF-*κ*B	IL-1*β*, TNF-*α*, IL-6, and MMPs	Proinflammatory signaling, ECM remodeling	IFN-*γ*, TNFR1/TNFR2
M2 macrophages	IL-4, IL-13	IL-10, TGF-*β*, and IL-1Ra	Resolution of inflammation	IL-10, TGF-*β*
Endothelium	Shear stress, NF-*κ*B	MCP-1	Leukocyte recruitment/activation	Selectins, selection-ligands, integrins, and CAMs
Antigen-presenting cells	DAMPs	NF-*κ*B, TNF-*α*, and lymphocyte activation	Detection of tissue injury	DAMPs, dendritic cells
TH1 lymphocytes	IL-2, IL-12	IFN-*γ*, TNF-*α*	Macrophage activation, endothelial activation	IFN-*α*
TH2 lymphocytes	IL-4	IL-4, IL-5, IL-9, IL-10, and IL-13	Unknown	IL-4, IL-13
Treg lymphocytes		IL-10, TGF-*β*	Antagonize TH1 cells and attenuate inflammation	IL-10, TGF-*β*
B1 lymphocytes		IgM NAb	Clearance of DAMPs	IgM NAb
B2 lymphocytes	IL-4, IFN-*γ*	IgG, IgE	Endothelial damage, macrophage uptake of DAMPs	IL-4, IFN-*γ*, IgG, and IgE
